# Wireless bioelectronic control architectures for muscle-powered biohybrid robotic systems

**DOI:** 10.1063/5.0339053

**Published:** 2026-10-13

**Authors:** Hiroyuki Tetsuka, Minoru Hirano

**Affiliations:** Bioinspired Systems Research-Domain, Toyota Central R&D Labs., Inc., 41-1 Yokomichi, Nagakute, Aichi 480-1192, Japan

## Abstract

Wireless bioelectronic interfaces are increasingly used to control tissue-engineered, muscle-powered biohybrid robotic systems. However, a unifying engineering framework linking device design to system-level control remains underdeveloped. Here, we formulate wireless control in biohybrid robotics as a coupled co-design problem integrating signal delivery, spatial selectivity, scalability, sensing and feedback, controller implementation, biological life support, and interface stability. We analyze three representative actuation and control strategies—wireless electrical stimulation, wireless optoelectronic stimulation, and neuromuscular integration—which operate in distinct regimes with characteristic trade-offs. Across these modalities, the tissue–device interface emerges as a key constraint governing the interplay among electrical or optical signal delivery, circuit performance, sensing, and biomechanical response. We outline practical design principles spanning electromagnetic field distribution, circuit architecture, actuator mechanics, multi-actuator addressing, and closed-loop control. Rather than treating neural organoids as an inevitable end point, we compare conventional electronic controllers, neuronal culture–silicon interfaces, and organoid–microelectrode array systems as alternative trajectories toward closed-loop autonomy. This system-level perspective provides design guidelines for stable, scalable, and application-driven biohybrid robotic systems.

## I. INTRODUCTION

Biohybrid robots embed living muscles—typically cardiac or skeletal muscle tissues—into highly pliable structures to create living machines capable of soft, adaptive behaviors like a living organism. These robots can mimic biological behaviors and functions such as life-like stimulus–response dynamics while retaining direct coupling to living cells and tissues. However, despite rapid progress in biohybrid robotics, achieving reliable, scalable, and application-relevant control remains a central challenge.

To contextualize the evolution in biohybrid robotics, key milestones can be interpreted as successive advances in controllability and system integration. Over the past two decades, the field has advanced from early proof-of-concept demonstrations of living muscle-powered microdevices and thin-film actuators to increasingly sophisticated biomimetic robots capable of swimming, walking, and multifunctional actuation.^1–19^ Figure 1 reframes it as an evolution of control strategies while emphasizing that future closed-loop systems can follow multiple architectural pathways rather than a single linear trajectory. Early studies emphasized muscle alignment control to improve anisotropic force generation.^2,3^ Subsequent work introduced optogenetic control to enable programmable remote activation of muscle tissues.^4,6,13^ More recent systems incorporated wireless bioelectronic and neuromuscular control, improving compatibility with enclosed environments and enabling selective actuation through multiplexed stimulation and neuron-mediated pathways.^5,7–11,15,16,19^ These approaches have often been optimized within individual modalities, leaving a need for a common engineering framework that connects device-level choices to system-level control performance.

**FIG. 1.**
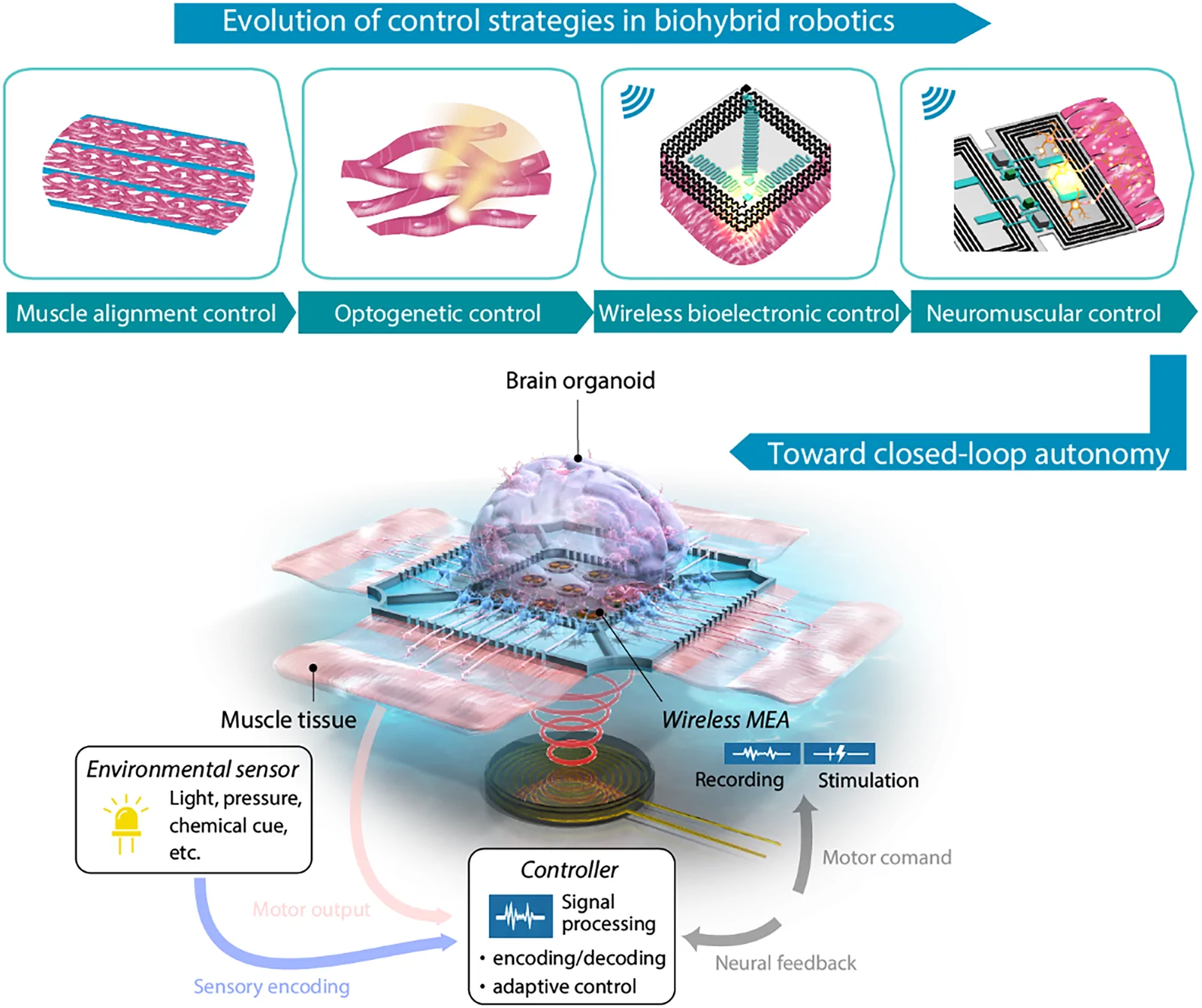
Evolution of control strategies in muscle-powered biohybrid robotics and possible pathways toward closed-loop autonomy. Top: representative historical progression from muscle alignment control to optogenetic control, wireless bioelectronic control, and neuromuscular control. This sequence is descriptive rather than a prescribed roadmap. Bottom: one prospective organoid–microelectrode array (MEA) branch in which environmental signals are encoded by a controller, delivered through a wireless MEA to a neural organoid, and decoded into motor commands that regulate a muscle array. Conventional electronic controllers and neuronal culture–silicon interfaces represent alternative closed-loop trajectories discussed in the text.

Several recent studies have provided comprehensive perspectives on the rapidly evolving field of biohybrid robotics. Kim *et al.* reviewed electrical, optical, and neuromuscular-junction-based stimulation strategies for muscle-driven biohybrid robots,^20^ while Wang *et al.* discussed the integration of soft electronic technologies for stimulation, sensing, and control.^21^ Quan *et al.* introduced a biophysics-informed framework for the design of biohybrid microrobots,^22^ and Li and Takeuchi summarized recent advances in contraction modeling, selective and closed-loop control, and onboard control strategies.^23^ Together, these studies have established important perspectives on stimulation modalities, bioelectronic interfaces, and biophysical design. However, the system-level relationships among wireless signal delivery, addressability, sensing and feedback, controller architecture, biological life support, and long-term interface stability remain less systematically discussed.

Here, we address this gap by formulating wireless control in muscle-powered biohybrid robotics as a coupled co-design problem. In this framework, system performance is jointly determined by interdependent factors, including signal delivery, spatial selectivity, scalability, sensing and feedback, controller architecture, biological life support, and interface stability. Existing control strategies can therefore be organized as distinct operating regimes with different trade-offs rather than as a simple hierarchy. By establishing this perspective, we aim to move beyond modality-specific optimization toward a system-level understanding that supports rational selection of stimulation, addressing, sensing, and controller architectures for a given application.

## II. WIRELESS CONTROL STRATEGIES

Table I summarizes the major wireless actuation and control directions considered here: wireless bioelectronics, wireless optoelectronics, neuromuscular integration, and one prospective organoid–wireless-MEA option. Wireless electrical stimulation offers the most direct route to untethered control, but its selectivity is often limited because stimulation is governed by field distribution and electrode geometry. Wireless optoelectronics improves spatial addressability through *μ*LED placement and patterned illumination but introduces optogenetic, optical-loss, thermal, and encapsulation constraints. Neuromuscular integration further expands controllability by exploiting biological signal transduction and multiplexed neural stimulation, at the cost of greater dependence on tissue maturation, synaptic reliability, and system complexity. These directions should therefore be interpreted as alternative design regimes with application-dependent trade-offs rather than as successive levels of technological maturity.

**TABLE I.** Comparative overview of wireless control strategies for untethered muscle-powered biohybrid robots and one prospective organoid–MEA controller option.

Direction	Signal path	Selectivity/addressability	Scale-up path	I/O	Closed-loop capability	Typical stability	Bottlenecks /	Refs.
(1) Wireless bioelectronics (electrical stimulation)	RF field -> receiver -> rectification/pulsing -> direct electrical pacing	Low-moderate; usually 1 global field or 1 local pacing site per receiver; field uniformity sets selectivity	Usually 1 receiver:1 output; scaling limited by coil coverage, standoff distance, and field nonuniformity	Usually stimulation-only; sensing/telemetry added separately	Open loop in representative systems; closed-loop needs added sensing + control	∼days-weeks in aqueous media	Media-appropriate electromagnetic and circuit co-design is essential; avoid overstimulation and electrochemical limits	7
(2) Wireless optoelectronics (wireless optical stimulation)	Wireless power -> micro-LED/uLED emission -> optogenetic activation	Moderate-high; 1-several emitter sites defined by emitter placement	Add emitters/addressing; limited by power budget, heat dissipation, and encapsulation	Mainly stimulation; communication/telemetry possible	Programmable open loop; closed-loop only when telemetry is added	Acute to short-term / multi-day; longer use limited by heat, encapsulation, and opsin stability	Optical loss, wavelength, irradiance duty cycle, emitter topology is a primary lever for selectivity	4, 13, 16, 19
(3) Neuromuscular integration	Wireless power/commands -> selective neural stimulation -> biological transduction to muscle	High; 2 actuator arrays independently addressed at 6.78 and 13.56 MHz	2 channels demonstrated; more via FDM/TDM, with higher spectral/control complexity	Mainly stimulation; recording can be added; biological transduction at the NMJ	High potential; steering/speed control shown, but integrated sensing is still limited	>150 s sustained pacing in one representative system; multi-week functional retention reported	Synapse modality and tissue maturation matter; multiplexing strategy shapes gait and steering	8–11, 15, 19
(4) Prospective option: Organoid + wireless MEA	Bidirectional MEA: stimulation input + recording output, ideally wireless in sealed systems	Very high; multi-site stimulation + recording; MEA systems can span tens-thousands of electrodes/channels	Limited by channel count, data bandwidth, decoding complexity, and chronic interface stability	Bidirectional by design: multi-channel recording + multi-site stimulation	Potentially compatible with adaptive closed-loop control; organoid-based control remains prospective	Weeks–months in wired/chronic organoid–MEA interfaces; long-term wireless stability not yet shown	Maturation and drift, life support, reproducibility, power/data bandwidth, chronic interface stability	

The concept of neural organoid–MEA integration should be viewed as one prospective controller architecture among several, rather than as an inevitable end point of biohybrid robotics. Relevant precedents include closed-loop learning and control in *in vitro* neuronal systems interfaced through MEAs,^24^ high-density organoid electrophysiology,^25^ cerebral organoid and assembloid interfaces enabled by kirigami electronics,^26^ organoid-intelligence frameworks for biocomputing,^27^ and size-adjustable MEA architectures that expand access to organoid activity.^28^ Conventional electronic controllers offer deterministic, low-latency, and reproducible control, whereas neuronal culture–silicon hybrids can introduce biological plasticity with comparatively simpler tissue maintenance and interfacing. Organoids may not only provide richer three-dimensional recurrent dynamics and multicellular organization but they also introduce greater challenges in maturation, reproducibility, diffusion and life support, chronic interfacing, and nonstationary decoding. Most organoid demonstrations remain wired; stable wireless, high-density, bidirectional organoid–MEA interfacing remains a major engineering challenge.

As shown in Fig. 2, the actuation modalities can be expressed within a common systems-level architecture. Key differentiators include (i) where the stimulus is generated, electrodes vs light emitters; (ii) whether biological transduction is leveraged, muscle-only vs neuromuscular pathways; (iii) whether bidirectional I/O is available for feedback; and (iv) which controller architecture is responsible for state estimation, decision-making, and adaptation. The latter function may be implemented by conventional electronics, hybrid neuronal culture–silicon systems, or, prospectively, organoid–MEA interfaces.

**FIG. 2.**
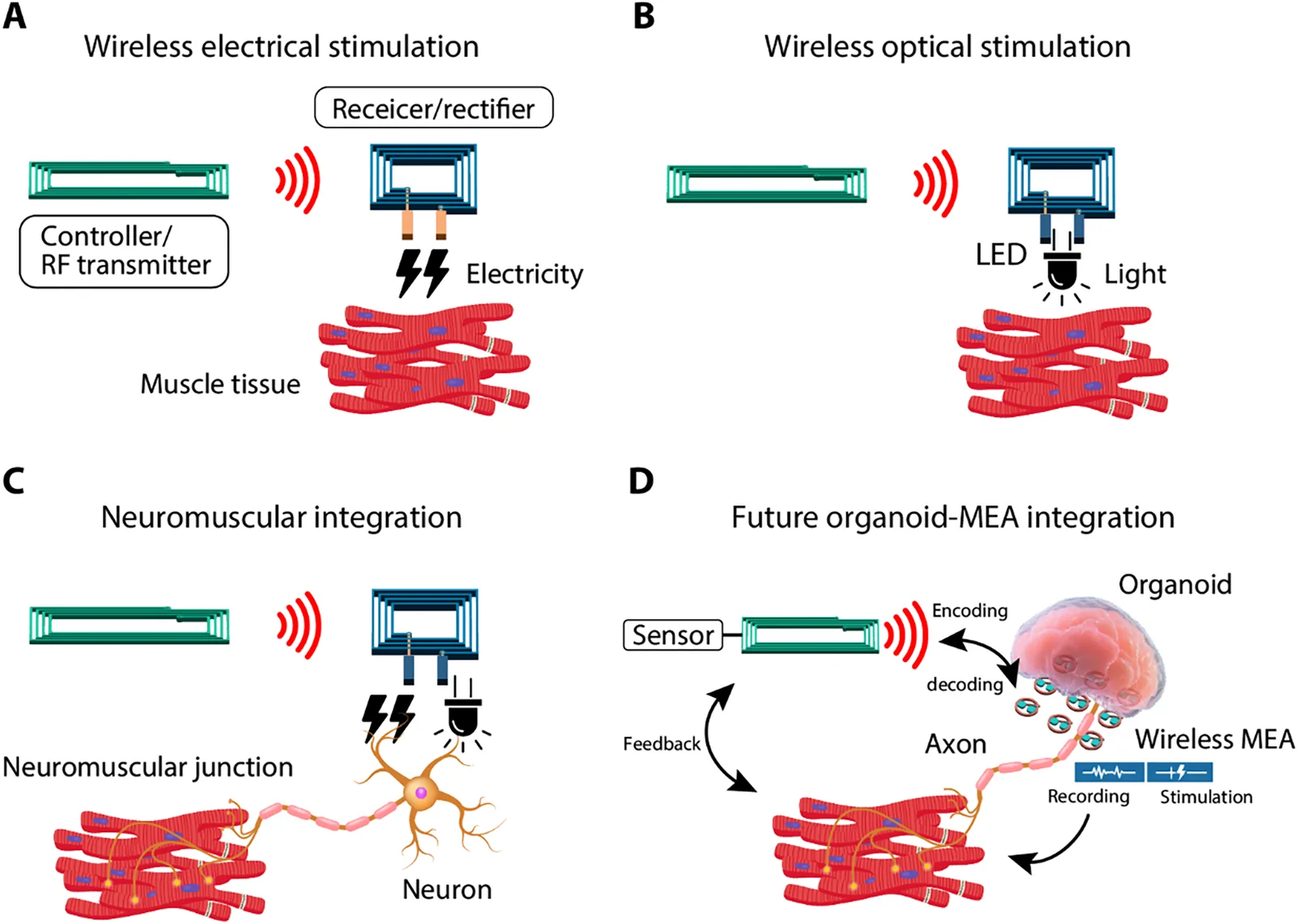
System-level architectures of wireless control in muscle-powered biohybrid robots across three established streams and one prospective organoid–MEA branch. (a) Wireless electrical stimulation: inductive radio frequency power transfer drives a receiver and rectifier that generate pulsed electrical output delivered through tissue-facing electrodes to directly pace muscle tissues. (b) Wireless optoelectronics: wireless power drives micro-light-emitting diode (*μ*LED) emitters to activate optogenetically modified muscle or neurons, enabling spatial selectivity through emitter placement. (c) Neuromuscular integration with selective wireless control: multiplexed excitation targets neural tissues coupled to muscle through neuromuscular or neurocardiac junctions, leveraging biological transduction to shape actuation and enable independent control of multiple actuators. (d) One prospective organoid–MEA direction: a bidirectional interface records neural activity and delivers stimulation patterns for closed-loop control. Conventional electronic and neuronal culture–silicon controllers provide alternative non-organoid implementations of the same closed-loop functions.

### A. Wireless bioelectronics

Wireless electrical stimulation is a core approach in wireless bioelectronics because it preserves direct electrical pacing without wired connections and remains one of the most established control routes in tissue-based biohybrid robotics.^29,30^ Its advantages are hardware simplicity and reliable pacing in closed and aqueous environments; its main limitations are signal selectivity, field nonuniformity, and electrochemical safety constraints at the tissue–electrode interface. One platform showed that wirelessly powered, stretchable, lightweight bioelectronics can be embedded directly into a hydrogel-based muscle tissue construct and remotely trigger locomotion.^7^ More recently, a miniaturized media-compatible wireless stimulator with a footprint of approximately 32 mm^2^ was integrated with a cardiomyocyte-powered biohybrid robot and used for external wireless pacing in culture medium.^31^ This miniaturization provides a practical route toward compact closed-system biohybrid robotics, while also highlighting the coupling and power-budget penalties associated with reducing receiver size.

A central design consideration is the co-design of the electric field distribution, circuit, and muscle tissue alignment in aqueous environments. The output voltage in culture media depends strongly on the surrounding dielectric environment, electrode impedance, and transmitter–receiver distance. The performance of the wireless bioelectronic platform drops rapidly with standoff distance.^7^ Electrical stimulation in conductive media is also sensitive to field nonuniformity and local current density. Electrode size, spacing, and placement should therefore be selected not only to exceed the stimulation threshold but also to avoid localized hotspots, overstimulation, or unstable pacing. This matters because the usable operating range is limited by stimulation thresholds, electrochemical safety constraints, and the mechanical bandwidth of the scaffold–tissue actuator, all of which have been identified as constraints in biohybrid robot design.^29,30^

Validation should be done in the intended operating environment. Radio frequency coupling efficiency and the resulting output waveforms can differ markedly between air and liquid. Coil geometry and driving conditions should therefore be optimized together to ensure reliable stimulation throughout the operating workspace while maintaining adequate safety margins and avoiding localized overstimulation. Even when adequate electrical pacing is achievable, controllability at the robot level is often limited by muscle relaxation dynamics and the viscoelastic response of hydrogel or scaffold composites. As a result, mechanical bandwidth sets the practical upper limit on actuation frequency and should be treated as a primary design constraint.

### B. Wireless optoelectronics

Wireless optical stimulation can be achieved using wireless optoelectronics by integrating light sources that activate optogenetically modified muscle tissue. Wireless optoelectronics extends remote control beyond direct electrical pacing by introducing spatially programmable optical stimulation. Its major strength is improved addressability, because stimulation sites can be defined by *μ*LED placement and illumination patterns rather than by electric-field distribution alone. At the same time, this advantage introduces additional constraints, including the need for optogenetic modification, optical attenuation in tissue and media, thermal management, encapsulation reliability, and long-term opsin stability.^32,33^ Previous studies have reported that optoelectronic robots incorporating battery-free *μ*LEDs support wireless control and real-time communication.^16^ Optical stimulation offers spatial selectivity through emitter placement and decouples actuation from direct electrode-tissue contact.

From a design perspective, effective optoelectronic control requires co-design between the biophysical properties of light-sensitive opsins and device hardware, including appropriate choices of wavelength, irradiance, and illumination strategy for the target tissue.^32^ Light wavelength, irradiance, and duty cycle are key parameters governing activation thresholds, temporal fidelity, and optical penetration. Because light is attenuated by both the culture medium and biological tissue, the irradiance delivered to the target cells can differ substantially from that generated at the emitter. Thermal management is therefore important, as prolonged illumination may induce biologically relevant heating that alters tissue function and compromises long-term operational stability.^33^ Potential phototoxic and photochemical effects associated with prolonged or high-intensity illumination should also be considered in the design of long-term systems.^32,33^ Recent studies have further integrated optogenetic myofibers with photoresponsive nanofiber scaffolds, demonstrating that optical stimulation can be designed together with the mechanical and responsive properties of the tissue-supporting structure rather than being considered solely in terms of emitter placement.^34^

### C. Neural integration

Neural integration provides an alternative to direct muscle pacing by enabling selective control of multiple actuators through biologically mediated signal-transduction pathways. A key advantage of this approach is the potential for enhanced selectivity through multiplexed neural pathways, although its performance depends on tissue maturation, synaptic reliability, and the long-term stability of neuromuscular function. Wireless frequency multiplexing provides one strategy for achieving such selective control by independently stimulating neural tissues coupled to different muscle actuators. In a representative system, two modulation signals were induced in separate resonant coils to generate voltage pulses that stimulated neural tissues, enabling independent modulation of two actuator arrays and control of robot speed and steering.^8^ The same work also showed that signal-transduction stability depended on synapse modality, with electrically coupled constructs sustaining controlled pacing longer than chemically coupled constructs.

More recently, neuromuscular junction-based biohybrid crawling robots integrated optogenetically modified motor neurons and skeletal muscle with onboard wireless μLED optoelectronics, enabling optical stimulation of neural tissue to modulate neuromuscular actuation.^19^ By shifting the site of stimulation from muscle to neurons, optoelectronic control expands the available actuation repertoire and reduces reliance on direct muscle pacing. This may be beneficial when repeated direct stimulation of muscle is undesirable because it may affect muscle integrity and limit long-term operation.

## III. CO-DESIGN PRINCIPLES

Remote control strategies for muscle-powered biohybrid robots should be selected according to application-specific design requirements. The choice of control architecture depends on signal delivery efficiency, selectivity, scalability, and interface stability, together with sensing requirements, controller implementation, biological life support, and the mechanical bandwidth of the living actuator.

Wireless electrical stimulation is particularly suitable when reliable actuation, simple hardware, and efficient power transfer are the primary requirements. As shown in Table I and Fig. 2(a), this approach is well suited to systems in which global pacing or stimulation at a single local site is sufficient for actuation. Its performance is strongly influenced by electric-field uniformity, electrode impedance, and the distance between the transmitter and receiver. Previous studies have reported monophasic outputs of approximately 2–9 V depending on device geometry and operating conditions, indicating that the effective operating range can decrease as wireless coupling becomes weaker. Reliable power delivery must therefore be maintained throughout the operating range while satisfying electrochemical safety limits and avoiding excessive local current densities.

Wireless optoelectronics is more suitable when spatially selective stimulation is required. As summarized in Table I and Fig. 2(b), multiple stimulation sites can be defined through the placement of optical emitters. This approach is particularly advantageous when localized stimulation provides a functional or biological benefit that is difficult to achieve by shaping the electric field alone.

A different control regime emerges when the goal extends beyond single-site actuation to selective control of multiple actuators or biologically mediated signal routing. Neuromuscular integration can provide functional selectivity that is difficult to reproduce with direct muscle pacing alone, and representative systems have demonstrated independent control of two actuator arrays using frequency-multiplexed wireless stimulation.^8^ Importantly, frequency-division multiplexing (FDM) and time-division multiplexing (TDM) are channel-access dimensions, whereas spatial addressing describes the physical localization of stimulation; they are therefore neither equivalent nor mutually exclusive and may be combined within the same system. Table II summarizes the main design considerations for these addressing strategies. FDM enables simultaneous activation when sufficient spectral separation between channels can be maintained, whereas TDM provides sequential control by allocating individual time slots to different channels. Spatial addressing instead defines stimulation locally through the physical arrangement of electrodes, coils, or optical emitters. In hybrid implementations, channel scalability must be considered together with power requirements, crosstalk, control latency, and the excitation and recovery dynamics of the biological actuators.

**TABLE II.** Design criteria for multi-actuator addressing in wireless muscle-powered biohybrid robots.

Addressing dimension	Activation pattern	Scalability / channel count	Dominant crosstalk	Latency	Power and biological constraints
FDM	Simultaneous activation across distinct carrier or resonance bands	Limited by usable spectral bandwidth, resonator Q, frequency spacing, and tolerance to detuning	Spectral overlap, resonance broadening, and medium- or motion-induced detuning	Low once a channel is selected	Parallel channels increase instantaneous power demand; receiver detuning and actuator bandwidth must be considered
TDM	Sequential time slots on a shared carrier or command path	Limited by slot duration, frame period, and minimum duty cycle required for each actuator	Temporal overlap, incomplete relaxation, and scheduling conflicts	Increases with number of channels and slot duration	Distributes power in time; slot timing must respect excitation, refractory, contraction, and relaxation dynamics
Spatial addressing	Local electrodes, coils, or *μ*LEDs define the stimulated region	Limited by site density, interconnect or addressing hardware, and available physical area	Electric-field spread, optical scatter, and mechanical coupling between adjacent actuators	Low to hardware-dependent	Power scales with number of simultaneously active sites; local current density, heating, and tissue damage set safety limits
Hybrid FDM/TDM + spatial	Concurrent or sequential activation with local physical targeting	Potentially highest addressability but with increased receiver, interconnect, and control complexity	Combined spectral, temporal, and spatial coupling	Architecture-dependent	Requires joint optimization of total power budget, addressing overhead, and biological/mechanical bandwidth

Finally, once the aim shifts from externally programmed stimulation to adaptive closed-loop behavior, bidirectional interfacing becomes indispensable. Closed-loop autonomy does not require a neural organoid specifically; it requires a controller that can receive state information, update an internal decision rule, and generate safe stimulation commands. Table III compares three controller families. Conventional electronic controllers provide the most deterministic and reproducible option, neuronal culture–silicon interfaces introduce biological plasticity with comparatively simple tissue geometry, and organoid–MEA systems may provide richer three-dimensional network dynamics but at the cost of greater variability and life-support burden. Suitable comparison metrics therefore include control error, response latency, adaptation time, decoding accuracy, I/O channel count and bandwidth, energy per control action, long-term uptime, baseline drift, recalibration frequency, and tissue-viability metrics.

**TABLE III.** Alternative controller architectures for closed-loop muscle-powered biohybrid robots.

Controller architecture	Distinctive functional strengths	Key limitations	Useful comparison metrics	Best-fit scenarios
Conventional electronic controller	Deterministic logic, low latency, high reproducibility, mature sensing and communication	Limited intrinsic biological plasticity; adaptation must be explicitly programmed	Tracking error, latency, energy per decision, computational load, uptime, robustness	High-reliability task control, fast feedback, long-duration operation with limited biological computation
Neuronal culture–silicon hybrid	Biological learning/plasticity with relatively accessible planar recording and stimulation	Culture variability, nonstationarity, limited tissue architecture, life-support requirement	Decoding accuracy, adaptation time, channel count, drift, recalibration interval, viability	Proof-of-concept adaptive control and studies of embodied neuronal learning
Organoid–MEA hybrid	Prospective access to three-dimensional recurrent dynamics, multicellular organization, and richer state space	Maturation and batch variability, diffusion and life-support constraints, difficult high-density interfacing, decoding drift	Decoding accuracy, effective dimensionality, adaptation time, channel count/bandwidth, drift, viability, chronic stability	Exploratory adaptive computation where biological dynamics are themselves a research objective

Application fit should guide this architectural choice. Muscle-powered biohybrid robots should not be assumed to outperform fully synthetic soft robots in general. Their clearest advantages arise when the living component itself provides a desired function, such as biologically matched compliance, direct interaction with cells or biochemical environments, remodeling or self-repair potential, and physiologically relevant actuation for disease models, drug testing, tissue-interaction studies, or soft devices operating in culture-like media. Fully synthetic soft robots remain preferable when high force, high speed, dry operation, wide temperature tolerance, or long maintenance-free deployment dominates the specification. For biohybrid systems, closed-loop control must therefore monitor both task performance and tissue state. Candidate sensors include strain and pressure sensors for mechanical output, impedance or electrophysiological interfaces for tissue state, temperature sensors for thermal safety, and pH, oxygen, or biochemical sensors for culture conditions.^21^ Life-support design must simultaneously maintain nutrient and oxygen delivery, temperature, pH and osmolality, sterility, waste removal, and media exchange. These constraints turn closed-loop control into a multi-objective problem that balances robotic performance against tissue viability.

## IV. CHALLENGES AND OPPORTUNITIES

These co-design constraints give rise to several challenges and opportunities. Miniaturization reduces coupling area and consequently the energy available at the interface, motivating higher-efficiency resonant receivers, improved field uniformity, and strategies that lower stimulation thresholds. For long-term use, electronics must remain reliable while tissue health is maintained, making packaging integrity, biological integration, biocompatibility, and life-support management central design requirements.^21,30,35^ Most current platforms operate in open loop with preset stimulation schedules. Reaching adaptive operation will require integrated sensing and feedback, including mechanical-state sensing, electrophysiology or impedance monitoring, and, where relevant, temperature and chemical-state measurements. These signals must be interpreted by a controller that accounts for both task-level error and biological constraints such as thermal exposure, stimulation safety, nutrient and oxygen availability, pH stability, and tissue fatigue. This broader architecture is consistent with electronic neural interfaces that combine tissue interfacing, sensing, stimulation, and signal processing.^36^

Future development is better described as a branching design space than as a linear roadmap toward organoid integration. One trajectory retains conventional electronic controllers and adds increasingly capable sensors, state estimation, and adaptive control algorithms. A second trajectory couples planar neuronal cultures to silicon interfaces to exploit biological learning or plasticity while preserving comparatively straightforward access for recording and stimulation. A third, more exploratory trajectory uses neural organoids as computational substrates connected through bidirectional MEAs. The appropriate branch depends on application-specific requirements for latency, reproducibility, adaptability, channel count, power, maintenance burden, and long-term stability. Organoid-based control is therefore presented here as one promising research direction rather than the inevitable end point of biohybrid robotics.

For the organoid branch specifically, the motivation comes from several converging advances: (i) closed-loop learning and control using *in vitro* neuronal systems interfaced via MEAs; (ii) high-density electrophysiological recordings of organoids sustained over weeks; (iii) chronic recordings from organoids and assembloids enabled by conformal electronics over months; and (iv) organoid-intelligence and reservoir-computing frameworks.^24–28,37^ Bidirectional I/O is a core requirement, but fully wireless MEA systems capable of stable, high-density, chronic stimulation and recording remain an engineering challenge with respect to power delivery, data bandwidth, encapsulation, and long-term biocompatibility.^35,36^ In addition, meaningful closed-loop autonomy cannot be inferred from spontaneous organoid activity alone. Functional control will require engineered encoding and decoding, task-specific training, and adaptive algorithms that explicitly manage nonstationarity caused by maturation, plasticity, and drift. Progress should therefore be evaluated using measurable system-level criteria such as decoding accuracy, response latency, adaptation time, chronic recording quality, stimulation safety, tissue viability, recalibration frequency, and long-term operational stability. Early demonstrations may benefit from low-dimensional tasks such as regulating actuation rate or locomotion speed in response to environmental variables before attempting high-dimensional autonomous behavior.

## V. CONCLUSION

In this work, we described a system-level engineering framework for wireless control of muscle-powered biohybrid robotic systems, in which performance is governed by the coupled design of signal delivery, selectivity, scalability, sensing and feedback, controller architecture, biological life support, and interface stability. We identified the transition from open-loop stimulation to closed-loop control as an important direction but emphasized that this transition can be realized through multiple controller architectures rather than a single linear path. Conventional electronics, neuronal culture–silicon interfaces, and organoid-integrated MEAs occupy different regions of the design space with distinct trade-offs in latency, reproducibility, adaptability, I/O complexity, maintenance burden, and chronic stability. Organoid-integrated bioelectronics remain a promising but exploratory option. Overall, this Perspective reframes wireless bioelectronic control as an application-driven system-level co-design problem and provides practical criteria for developing stable, scalable, and increasingly autonomous biohybrid robotic systems.

## Data Availability

The data that support the findings of this study are available within the article.
